# The Causes and Consequences of Changes in Virulence following Pathogen Host Shifts

**DOI:** 10.1371/journal.ppat.1004728

**Published:** 2015-03-16

**Authors:** Ben Longdon, Jarrod D. Hadfield, Jonathan P. Day, Sophia C. L. Smith, John E. McGonigle, Rodrigo Cogni, Chuan Cao, Francis M. Jiggins

**Affiliations:** 1 Department of Genetics, University of Cambridge, Cambridge, United Kingdom; 2 Institute of Evolutionary Biology, University of Edinburgh, Edinburgh, United Kingdom; 3 Department of Ecology, University of São Paulo, São Paulo, Brazil; Stanford University, UNITED STATES

## Abstract

Emerging infectious diseases are often the result of a host shift, where the pathogen originates from a different host species. Virulence—the harm a pathogen does to its host—can be extremely high following a host shift (for example Ebola, HIV, and SARs), while other host shifts may go undetected as they cause few symptoms in the new host. Here we examine how virulence varies across host species by carrying out a large cross infection experiment using 48 species of Drosophilidae and an RNA virus. Host shifts resulted in dramatic variation in virulence, with benign infections in some species and rapid death in others. The change in virulence was highly predictable from the host phylogeny, with hosts clustering together in distinct clades displaying high or low virulence. High levels of virulence are associated with high viral loads, and this may determine the transmission rate of the virus.

## Author Summary

Many emerging infectious diseases are the result of a host shift, with the pathogen jumping into the new host from another species. Virulence—the harm a pathogen does to its host—can be extremely high following a host shift (for example HIV, SARs and Ebola), while other host shifts may go undetected as they cause few symptoms in the new host. We have found that variation in virulence following host shifts can be extremely large and were highly predictable from the host phylogeny, with hosts clustering together in distinct clades displaying high or low virulence. These changes in virulence result from changes in viral load, and therefore the transmission potential of the virus. This suggests there is no clear rule to predict whether a pathogen will be virulent in a novel host. However, it does suggest a simple rule of thumb may be that if a pathogen causes high levels of virulence in any given host species, it will typically cause similar levels of virulence in closely related hosts.

## Introduction

Virulence—which we define as the harm a pathogen does to its host—can sometimes dramatically increase when pathogens shift to infect new host species, resulting in new and devastating outbreaks and epidemics [1,2]. For example, in bats, Ebola virus appears to be largely asymptomatic, but it is frequently fatal when it crosses the species barrier into humans and other primates [3,4]. Similarly Henipaviruses appear to be non-pathogenic in pteropoid bats, but can cause high levels of mortality in livestock and humans [5,6].

Despite the importance of these virulence changes, we have little understanding of what determines changes in virulence following a host shift. Historically, it was thought that novel host-parasite associations result in high levels of virulence and that long-term host-parasite interactions lead to the pathogen evolving towards avirulence [7]. However, despite several high profile examples of host shifts resulting in extremely high virulence (see above), there may be a large ascertainment bias in detecting novel infections following hosts shifts, with only the most virulent likely to be detected and many benign infections going unnoticed [8]. Likewise, it has been shown that long term coevolution does not necessarily lead to avirulence [9–12].

To predict how virulence will change following a host shift, we need to understand what causes virulence. Virulence is sometimes thought to be a direct consequence of pathogen replication, with greater levels of replication causing greater amounts of damage to the host [13]. However, virulence and parasite replication can be decoupled [14], especially if hosts mount an inappropriate immune response against novel pathogens [15]. Similarly, virulence can result from infection of a tissue type that has no adaptive value to the pathogen [14,16]. For example, virulence in bacterial meningitis is a result of infection of the central nervous system, and has no relation to transmission potential, or infection of other tissues, where it causes no symptoms [16].

Novel host-parasite interactions have not been under direct selection [17], so the level of pathogen virulence is likely to be maladaptive not only for the host but also the pathogen. This contrasts with natural associations where pathogens have been selected to optimise their virulence in order to maximise their between-host transmission potential [9], and hosts have been selected to reduce the harm caused by their pathogens (to ‘tolerate’ infection) [13,18–22], although one-sided host resistance may evolve in cases where the pathogen-causes a dead end infection [17]. The lack of adaptation in novel host-pathogen associations may explain dead-end chains of transmission seen in some emerging diseases [23]. Here maladaptive levels of virulence may mean the host has less chance of transmitting the pathogen [24], potentially preventing it from becoming established in the population. The classic example of the myxoma virus highlights how virulence may be maladaptive following a host shift. For example, the myxoma virus is relatively benign in South American *Sylvilagus* rabbits, but it was transferred to European rabbits (*Oryctolagus cuniculus*) as a biological control agent where it initially had case-mortality rates as high as 99.8% in Australia and drove many populations to near extinction [20,25]. However, following its introduction to European rabbit populations in Australia, case-mortality rates dropped rapidly. This was due to the spread of attenuated virus strains that had higher rates of transmission due to infected hosts surviving, and therefore transmitting the virus, for longer [20,25].

Whilst we know little about how virulence changes following a host shift, the ability of pathogens to infect novel hosts has been more widely studied [26–28]. A number of studies have revealed that the host phylogeny is an important factor in determining the susceptibility of novel hosts [29–35]. The first effect of the host phylogeny is that species closely related to the natural host of a pathogen tend to be more susceptible. This is thought to be because parasites evolve specialised adaptations to their natural host, such as binding to host receptors, avoiding immune responses or utilising host resources, and these break down if the environment provided by the novel host is too different [28]. Secondly, independent from the genetic distance from the pathogen’s natural host, closely-related groups of hosts may have similar levels of susceptibility. Such patterns could arise due to the loss or gain of immune or cellular components in different lineages (e.g. [36,37]), resulting in the host phylogeny being a patchwork of host clades varying in their susceptibility.

We have used a large cross-infection experiment to understand how virulence changes following a host shift. We infected 48 species of *Drosophilidae* with Drosophila C virus (DCV) and measured virulence, the change in viral load, and the transmission potential of the virus. DCV is a positive sense RNA virus in the family *Discistroviridae* that was isolated from *D*. *melanogaster* and naturally infects *D*. *melanogaster* and *D*. *simulans* in the wild [38]. The full host range of DCV in the wild is unknown, but it can infect other species of Drosophilidae in the laboratory [39] and can replicate when injected into other dipterans and a moth [40]. DCV causes a reduction in metabolic rate, lowers activity levels, causes intestinal obstruction, lowers the pH of the haemolymph and can ultimately result in death [41–44]. DCV is targeted by antiviral RNAi and a JAK-STAT pathway dependent immune response [45,46]. Here, we have investigated how the host phylogeny determines variation in virulence between species, and the relationships between virulence, viral load and the transmission potential of the virus.

## Methods

### Virus production

DCV was produced in Schneider’s Drosophila line 2 (DL2) cells [47] as described in [48]. The DCV strain used was isolated from *D*. *melanogaster* collected in Charolles, France [49]. Cells were cultured at 26.5°C in Schneider’s Drosophila Medium with 10% Fetal Bovine Serum, 100 U/ml penicillin and 100 μg/ml streptomycin (all Invitrogen, UK). Cells were then freeze-thawed twice to lyse cells and centrifuged at 4000g for 10 minutes at 4**°**C to remove any cellular components or bacteria. The resulting virus suspension was aliquoted and frozen at -80**°**C. Uninfected cell culture for control sham inoculations was produced by growing DL2 cells as for virus production but DCV was replaced with Drosophila Ringer’s solution [50]. To calculate infectivity of the virus, serial dilutions of virus from 10^-1^ to 10^-12^ were carried out in Schneider’s medium, and each dilution was added to 8 wells of a plate of DL2 cells. After 7 days the wells were examined and classed as “infected” when cell death and cytopathic effects were clearly visible. The Tissue Culture Infective Dose 50 (TCID_50_) was calculated by the Reed-Muench end-point method [51].

### Inoculating flies

48 species of flies which share a common ancestor ~20–50mya were used in this study [52]. All fly stocks were reared at 22**°**C and 70% relative humidity. Stocks of each fly species were kept in 250ml bottles at staggered ages, and each day freshly eclosed flies were sexed, females were removed, and males were placed on cornmeal medium for 2 days before inoculation. The food medium used for rearing and details of the fly stocks and food recipes used can be found in Table A in S1 Text.

To inoculate flies, a 0.0125 mm diameter stainless steel needle (26002–10, Fine Science Tools, CA, USA) was bent ~0.25 mm from the end, dipped in DCV (TCID_50_ = 4.64×10^9^) or control solution (uninfected cell culture medium), and the bent part of the needle pricked into the pleural suture on the thorax of flies. Flies were then placed into vials of cornmeal medium and kept at 22**°**C and 70% relative humidity. These conditions were chosen as they were suitable to maintain all 48 species and carry out infection assays.

As a measure of virulence we recorded mortality after infection. The number of dead flies was counted each day for 20 days and flies were transferred onto fresh medium every 3 days to minimise mortality unrelated to infection. To measure the change in viral load, half of the flies were snap frozen immediately after inoculation in liquid nitrogen as a reference sample to control for relative dose, and the rest were kept for 2 days before being snap frozen in liquid nitrogen. The day 2 time-point was chosen based on time-course data for 10 host species (Figure A in S1 Text), with viral load beginning to plateau after this time. Frozen flies were then homogenised in Trizol reagent (Invitrogen) and stored at -80**°**C for later RNA extractions.

The mortality inoculations were carried out over a period of 3 days, with the aim of completing a control and virus treatment biological replicate for each fly species each day; i.e. each fly species was included in 2 blocks each day of the experiment. Treatment (virus or control) and the order in which fly species were inoculated was randomized between blocks. The inoculations for measuring the change in viral load were carried out over 6 days, with each species being inoculated each day. Treatment (frozen immediately or on day 2 post infection) and the order the fly species were infected was randomized each day.

In total we measured survival or the change in viral load in 12, 276 flies. In the mortality treatment we measured survival in a mean of 22 flies per replicate (range across species means = 7–25 flies). Out of the 48 species, 41 had 6 biological replicates, 2 had 5 biological replicates, 2 had 4 biological replicates and 3 had 3 biological replicates for the mortality treatments. On average we quantified viral load in a pool of 23 flies per replicate (range across species means = 10–25 flies) with the aim of producing 3 pairs of day 0 and day 2 biological replicates. Out of the 48 species, 45 had 6 biological replicates and 3 had 4 biological replicates for the viral load measurements.

### Other factors

Fly stocks were tested for *Wolbachia* bacterial endosymbionts using PCR primers that amplify the *wsp* gene [53] prior to the experiment. *Wolbachia* have been shown to provide resistance to DCV [47,54]) and so only *Wolbachia-*free species were used; 43 species were naturally Wolbachia free, 5 species were derived from antibiotic treated lines. Species that had a pre-existing DCV infection were also excluded from the experiment.

We also checked that the results were not affected by differences in body size between the species. Wing length is commonly used as a body size measure in *Drosophila* and strongly correlates with thorax length [55,56]. Wings were removed from ethanol-stored flies collected at the start of the experiment, and photographed under a dissecting microscope. On average we measured wings from 34 individuals per species (range = 13–47). The length of the IV longitudinal vein from the tip of the proximal segment to where the distal segment joins vein V [57] was measured (relative to a standard measurement) using ImageJ software (v1.48) [58].

### Measuring the change in viral load

We measured the change in RNA viral load using qRT-PCR. The viral RNA load was expressed relative to the endogenous control housekeeping gene *RpL32* (*Rp49*). We sequenced *RpL32* for each species and designed specific *RpL32* primers for each species in two conserved regions as described in [30]. We found the DCV primers we used amplified multiple products for *D*. *tropicalis*, so a different primer pair was used for this species (as we are measuring the change in relative viral load this should not affect the results). Primer efficiencies were calculated using a dilution series and were close to 100% (DCV: 104%; alternative DCV primers for *D*. *tropicalis*: 97%; *RpL32*: mean = 106%; range of 98–112%). DCV primers were DCV qPCR 599F (5’-GACACTGCCTTTGATTAG-3’) and DCV qPCR 733R (5’-CCCTCTGGGAACTAAATG-3’)[48], the alternative *D*. *tropicalis* DCV primers were DCV qPCR 3477F (5’-TTCTTGGTTAGGTCGATTCTTTT-3’) and DCV qPCR 3611R (5’-AATTCTTCGGCTCCAGCTTC-3’).

Total RNA was extracted from Trizol homogenised flies, reverse-transcribed with Promega GoScript reverse transcriptase (Promega) and random hexamer primers, and then diluted 1:10 with nuclease free water. The qRT-PCR was performed on an Applied Biosystems StepOnePlus system using Sensifast Hi-Rox Sybr kit (Bioline) with the following PCR cycle: 95**°**C for 2min followed by 40 cycles of: 95**°**C for 5 sec followed by 60**°**C for 30 sec. Two qRT-PCR reactions (technical replicates) were carried out per sample with both the viral and endogenous control primers. Each qRT-PCR plate contained four standard samples, and all experimental samples were split across plates in a randomised block design. A linear model was used to correct the cycle threshold (Ct) values for differences between qRT-PCR plates. To estimate the change in viral load, we first calculated Δ*Ct* as the difference between the cycle thresholds of the DCV qRT-PCR and the endogenous control. The viral load of day 2 flies relative to day 0 flies was then calculated as 2^-ΔΔ*Ct*^, where ΔΔ*Ct* = Δ*Ct*_*day0*_ –Δ*Ct*_*day2*_, where Δ*Ct*_*day0*_ and Δ*Ct*_*day2*_ are a pair of Δ*Ct* values from a day 0 biological replicate and a day 2 biological replicate for a particular species. We note that calculating the change in viral load without the use of the endogenous control gene gives equivalent results (Spearman’s correlation between viral load calculated with and without endogenous control = 0.97, *P*<0.001).

### Host phylogeny

The host phylogeny was inferred using the *COI*, *COII*, *28S rDNA*, *Adh*, *SOD*, *Amyrel* and *RpL32* genes. We downloaded all the available sequences from Genbank, and attempted to sequence *COI*, *COII*, *28S rDNA*, *Adh* and *Amyrel* in those species from which they were missing, as described in [30]. This resulted in sequence for all species for *COI*, *COII* and *RpL32* and partial coverage for the other genes (34 out of 336 species-locus combinations were missing, S1 Table). The sequences of each gene were aligned using ClustalW (alignments and Genbank accession numbers for sequences used are available in an online repository http://dx.doi.org/10.6084/m9.figshare.1112749 and as S1 Dataset). To reconstruct the phylogeny we used BEAST (v1.8.0) [59] as this allows construction of an ultrametric (time-based) tree using a relaxed molecular clock model. Using ultrametric trees assumes that rates of evolutionary change (virulence and viral load) are proportional to time (rather than proportional to the rate of sequence evolution in the genes used to construct the phylogeny). The genes were partitioned into 3 groups each with their own substitution and molecular clock models. The three partitions were: mitochondrial (*COI*, *COII*); ribosomal (*28S*); and nuclear (*Adh*, *SOD*, *Amyrel*, *RpL32*). Each of the partitions used an HKY substitution model [60] (which allows transitions and transversions to occur at different rates) with a gamma distribution of rate variation with 4 categories and estimated base frequencies. Additionally the mitochondrial and nuclear data sets were partitioned into codon positions 1+2 and 3, with unlinked substitution rates and base frequencies across codon positions. Empirical studies suggest that HKY models with codon partitions are a good fit for most protein-coding data sets [61]. A random starting tree was used, with a relaxed uncorrelated lognormal molecular clock and we used no external temporal information, so all dates are relative to the root age. The tree-shape prior was set to a speciation-extinction (birth-death) process. The BEAST analysis was run twice for 100 million MCMC generations sampled every 1000 steps. The two MCMC processes were examined using the program Tracer (v1.6) [62] to ensure convergence and adequate sampling. The two runs were combined using the programme LogCombiner (v1.8). A maximum clade credibility tree was created following a 10% burnin using TreeAnnotator (v1.8) and visualised using FigTree (v. 1.3.1) [63] (Tree nexus file http://dx.doi.org/10.6084/m9.figshare.1112749).

### Statistical analysis

We used a phylogenetic mixed model to examine the effects of host species relatedness [64–66] using the MCMCglmm R package ([67], R Foundation for Statistical Computing, Vienna, Austria). We used a trivariate formulation with mortality of the controls, mortality of the virus infected flies and viral load as response variables. Viral load was treated as Gaussian, whereas the mortality data were treated as longitudinal binomial data as in event history analysis [68]. The daily binomial counts are the number of flies that died and survived between successive days, with days where both counts are zero (i.e. all flies in the vial died the previous day) omitted as they are uninformative. The mortality model is equivalent to a proportional odds survival analysis (with censoring) [69]. Mortality and viral load data are provided in S2 Dataset.

The model structure is similar to the trivariate model outlined in [30]:

$$\eta_{thij}=\beta_{1:t}+distance\beta_{2:t}+wingsize\beta_{3:t}+\delta (age\beta_{4:t}+\beta_{5:t}age2+\beta_{6:t}food+u_{v:thi})+u_{p:th}+u_{s:th}+e_{thij}$$

where *η*_*thij*_ is the *j* ^*th*^ linear predictor for the *i* ^*th*^ biological replicate of host species *h* for trait *t*. *β*_*v*_ For the viral load trait, *j* always equals 1, but for the mortality traits *j* takes on values 1 to n, where n is either 20 days (the length of time over which mortality was recorded) or the number of days it took for all flies in a vial to die if this occurred in less than 20 days.

*β* are fixed effects with *β*_*1*_ being the intercepts for each trait and *β*_*2*_ and *β*_*3*_ the linear effects of wing size and genetic distance from *D*. *melanogaster* respectively. For the two mortality traits, separate linear and quadratic age effects were also included for each treatment, as was a linear effect of days since the flies were transferred onto fresh food medium: *δ* is one if the trait is a mortality trait and zero otherwise

*u* are random effects for vial (*v*—mortality traits only), phylogenetic species effects (*p*) and non-phylogenetic species effects (*s*) and *e* are residuals. The random effects (and residuals) are assumed to be multivariate normal with zero mean and covariance structure **V** ⊗ **A** for phylogenetic effects or **V** ⊗ **I** otherwise. **I** is an identity matrix, **A** the phylogenetic relatedness matrix and **V** a matrix of estimated variances and covariances. For the phylogenetic and non-phylogenetic species effects **V** is a 3x3 covariance matrix describing the (phylogenetic and non-phylogenetic) inter-specific variances in each trait and the inter-specific covariances between them. For the residuals **V** is also a 3x3 matrix, but for the vial effects that are only defined for the two mortality traits, **V** is a 2x2 covariance matrix. The off-diagonal elements of **V** for the residual and vial effects were set to zero because the covariances between traits at these levels are not estimable by design.

The linear predictor defines the expected value of the response *E[y*_*thij*_]/*w*_*thij*_ = *g* ^*-1*^ *(η*_*thij*_) where *g* ^*-1*^ is the inverse-link function, and *w*_*thij*_ is a weight. For the viral load trait *g* ^*-1*^ is the identity function and the weights are one. For the mortality traits *g* ^*-1*^ is the inverse logit function *(exp(η*_*thij*_))/(*1+ exp(η*_*thij*_)) and the weights are the number of flies alive at the end of the previous observation (or for *j* = 1 the number of flies initially present).

Diffuse independent normal priors were placed on the fixed effects (means of zero and variances of 10^8^). Parameter expanded priors were placed on the (co)variance matrices resulting in scaled multivariate F distributions which have the property that the marginal distributions for the variances are scaled (by 1000) F_1,1_. The exceptions were the residual variances for which an inverse-gamma prior was used with shape and scale equal to 0.001. The MCMC chain was ran for 1300000 iterations with a burn-in of 300000 and a thinning interval of 500.

We confirmed the results were not sensitive to the choice of prior by also fitting models with inverse-Wishart and flat priors for the matrices describing the phylogenetic and non-phylogenetic species effects (described in [30]), which gave qualitatively similar results (data not shown). We also ran models with only phylogenetic or non-phylogenetic species effects, which gave equivalent results in all instances (data not shown). To confirm our results were robust to different analysis methods we also analysed the mortality data by calculating the proportion of flies dead on each day of the experiment, taking the mean across all the days (which will be equivalent to the area under the survival curve), and arcsine square-root transforming this number. We then used this together with the change in viral load as responses in a trivariate model as described above. This gave very similar results to the proportional odds model (data not shown). Finally, we also ran models where the logit-linear dependency varies across species (variable age-dependent mortality), and these models also gave very similar results (data not shown).

### Transmission of DCV

In order to understand how DCV is transmitted, and how viral load might relate to transmission, we performed two additional transmission experiments. In the first experiment a single infected adult fly of the focal species was housed with adult sentinel flies (from a *D*. *melanogaster* isogenetic line) and the infection status of the sentinels (adult-adult transmission) and their offspring (adult+parent-offspring transmission) assayed. In the second experiment corpses of single adult flies (of the focal species) that died through DCV infection were housed with sentinel eggs, and the infection status of the emergent adult sentinels assayed (dead adult-embryo/larvae transmission). In both cases, we examined the transmission rates and tested for a correlation between transmission success and viral load from the previous experiment, which was measured 2 days after inoculation. To ensure that this viral load was correlated with the viral load in corpses of flies that died from a DCV infection, we also measured viral load in corpses for a subset of species. Full methods (S1 Text) and data (S1 and S3 Datasets) can be found in the Supporting Information.

## Results

### Host shifts result in large changes in virulence

**Fig 1:**
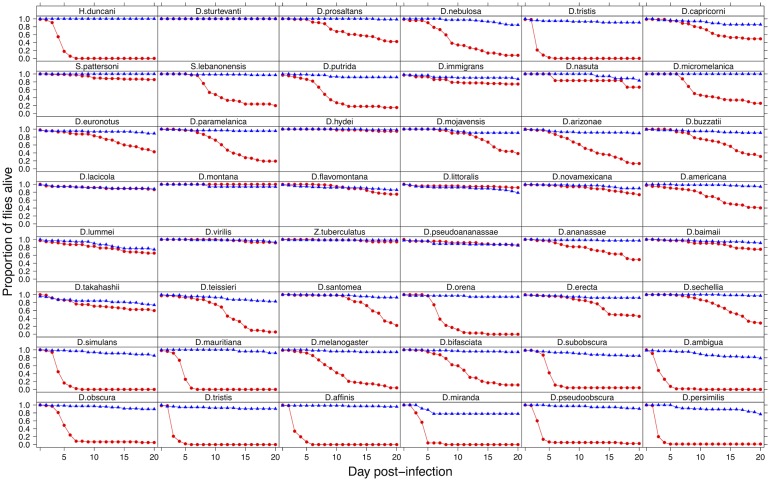
Mortality in 48 species of Drosophilidae after infection with DCV. Virus infected flies are red circles and control flies are blue triangles. Panels are ordered as in the tip order in Fig. 2.

To investigate the effect of switching hosts on virulence, we inoculated 2967 flies from 48 species of Drosophilidae with DCV and recorded mortality (virulence) for 20 days. We found dramatic differences in the virulence of DCV when it infects different host species (Fig. 1). In some species it appears to be a largely benign infection, with the virus causing no increase in mortality over 20 days. At the other extreme, ~90% of *D*. *persimilis* and *D*. *pseudoobscura* flies are killed by day 4 post-infection.

**Fig 2:**
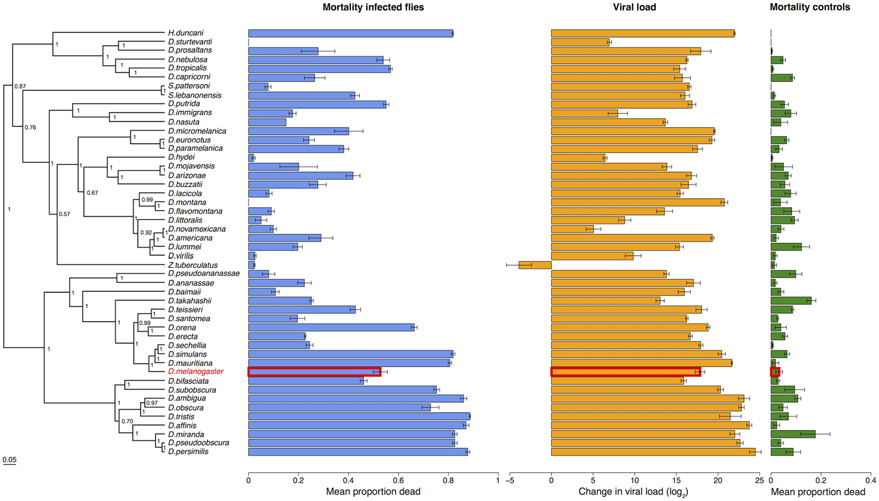
Phylogeny of host species, virulence and viral load. Virulence is measured as mean mortality of virus infected flies across all the days of the experiment. Viral load is the change in viral load between day 0 and day 2 post infection (see Figure B in S1 Text for data at each time point). *D*. *melanogaster*, the species DCV was isolated from, is highlighted in red. The common ancestor of the host species is estimated to be ~40 million years ago [52,84]. Graphs show means and standard errors. Node labels on the phylogeny are posterior supports, the scale bar is the number of substitutions per site.

To confirm that these differences reflect the virulence of the virus rather than intrinsic differences in the survivorship of the different species, alongside the virus infections we also inoculated 2993 flies from the 48 species with a control solution. There was far less mortality in the controls than the virus infected flies (range of mortality for controls: 0–18% and infected: 0–89%, see Figs. 1 and 2). Nonetheless, there was significant inter-specific variation (the sum of both phylogenetic and non-phylogenetic variation) in control mortality, which accounted for 43% of the between-vial variation in mortality (95% CI = 15%-74%), but this was not correlated with mortality in infected flies (inter-specific correlation = -0.08, CI = -0.53, 0.34). We also found no significant relationship between wing size and the mortality of infected flies (-0.44, 95% CI = -2.56, 1.78).

### Virulence following host shifts is determined by the host phylogeny

To examine how the host phylogeny influences changes in virulence, we reconstructed the phylogeny of our 48 Drosophilidae species using seven genes. The resulting tree is broadly consistent with previous studies of these taxa [70], with the close phylogenetic relationships being generally well supported (Fig. 2).

Because DCV was isolated from and naturally infects *D*. *melanogaster* [38], we examined whether virulence was affected by genetic distance from this host species. We found there was no significant change in virulence with distance from *D*. *melanogaster* (Fig. 2; slope = -0.76, 95% CI = -3.37, 1.62).

Despite there being no effect of genetic distance from *D*. *melanogaster* on virulence, there is a striking pattern of high- and low-virulence hosts clustering together on the phylogeny (Fig. 2). We used a phylogenetic mixed model to partition the inter-specific variance in mortality into that which is explained by a Brownian motion model of evolution on the host phylogeny (*v*_*p*_), and a species-specific component independent of the phylogeny (*v*_*s*_). We then calculated the proportion of between-species variance in the treated group that is explained by the host phylogeny (*v*_*p*_/(*v*_*p*_ + *v*_*s*_), which is related to Pagel’s lambda or phylogenetic heritability [65,66,71]. 75% of the between-species variation in virulence is explained by the host phylogeny (95% CI = 48%-97%).

**Fig 3:**
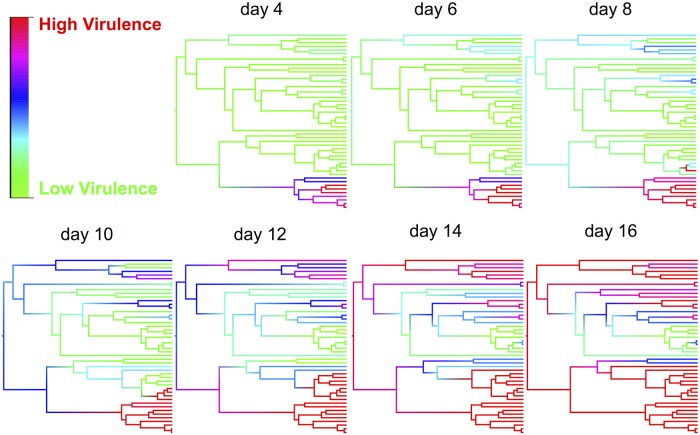
Ancestral state reconstructions of virulence for various time points post infection. Ancestral states were estimated from the phylogenetic mixed model for each node and then plotted as colour gradients across the tree. Colours represent the virulence (proportion of flies dead), with red representing the highest level of virulence and green as the lowest level of virulence at that time point. The ordering of tips on the phylogeny is as in Fig. 2. See a video of the change in virulence over time here: http://dx.doi.org/10.6084/m9.figshare.1192890

The effect of the host phylogeny on virulence can be clearly seen by using this model to reconstruct mortality rates across the phylogeny on different days post infection (Fig. 3 and video http://dx.doi.org/10.6084/m9.figshare.1192890). Early in the course of infection significant mortality only occurs in a single small clade of species (obscura group), but as time progresses other clades start to experience high mortality, and by day 16 most of the survivors belong to a single clade of species (virilis group) where virulence is relatively low (Fig. 3).

### Virulence following host shifts is strongly correlated to viral load

**Fig 4:**
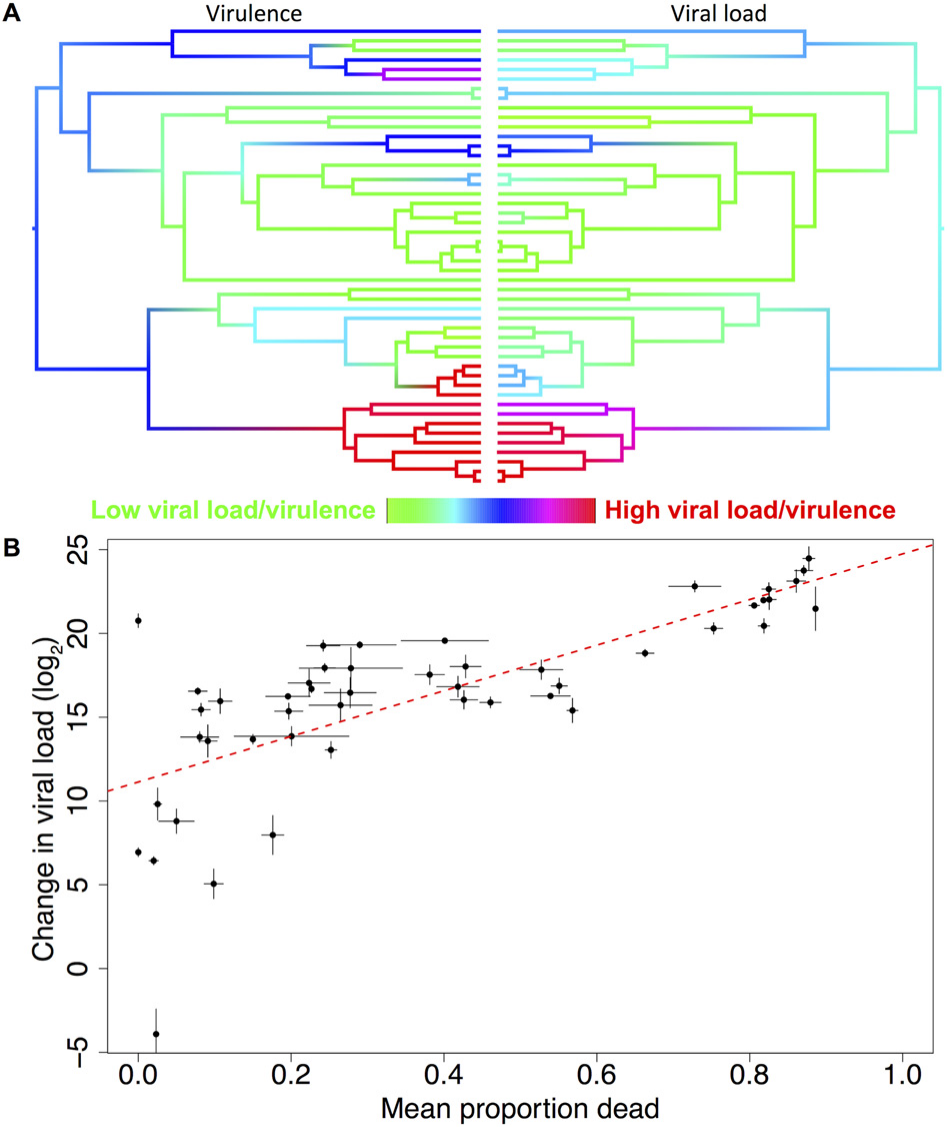
Correlation between viral load and virulence. (**a**) Mirrored phylogenies showing ancestral state reconstructions of virulence at day 10 post-infection (left) and viral load (right). Colours represent the virulence (proportion of flies dead) or viral load with red representing the highest levels and green the lowest. The ordering of tips on the phylogenies is as in Fig. 2. (**b**) Raw viral load and virulence (mean proportion of flies dead across all days in the experiment) data for 48 host species, the trend line is estimated from a linear model, error bars show standard errors. The inter-specific correlation is 0.69 (95% CI = 0.45, 0.88).

To measure viral load we inoculated 6316 flies from all 48 species and measured the change in viral RNA load by qRT-PCR (Figure B in S1 Text). Similar to virulence, there are extremely large differences in viral load between species, with the total inter-specific variance explaining 91% of the variance in viral load. Indeed, we found roughly a billion times the amount of viral RNA in the most susceptible host species compared to the most resistant species (Fig. 2). This was unrelated to the size of the flies, with no significant effect of wing size on viral load (slope = 0.15, 95% CI = -6.04, 6.27),

Species with high viral loads tend to cluster together on the phylogeny (Fig. 4). This was confirmed by fitting a phylogenetic mixed model, where 67% (95% CI = 33%-93%) of the between-species variation in viral load is explained by the host phylogeny. As was the case for virulence, we found there was no significant change in viral load with distance from *D*. *melanogaster*, the species from which the virus was isolated (slope = -2.01, 95% CI = -7.90, 7.08).

Variation in virulence could either be a consequence of different pathogen loads, or due to similar pathogen loads causing different levels of harm to their hosts. To separate these hypotheses, we examined the relationship between virulence and viral load. We found strong positive phylogenetic and non-phylogenetic correlations between virulence and viral load (Fig. 4, phylogenetic correlation = 0.76, 95% CI = 0.44, 0.99; non-phylogenetic correlation = 0.47, 95% CI = 0.05, 0.83). There is no correlation between viral load and the mortality that occurred in the mock-infected control flies (inter-specific correlation = 0.06, 95% CI = -0.38, 0.52).

### Virulence and transmission rate in novel hosts

We investigated the efficiency of different routes of transmission by allowing uninfected sentinel *D*. *melanogaster* to come into contact with infected flies (see supplementary information). We then tested pools of sentinel flies for the presence of DCV. In our assay we could not detect any adult to larval transmission in the 8 species we tested, despite these species all having high viral loads (*n* = 21 biological replicates). This suggests shedding of the virus is not a major source of infection for larvae. Next, we measured transmission between adult flies, and detected transmission in 11% of vials (*n* = 107 biological replicates across 38 species). Finally, we tested whether transmission occurred from the corpses of dead flies to embryos or larvae, and found that this was a highly efficient transmission route, with 80% of vials of sentinel flies testing positive for DCV infection (*n* = 44 biological replicates across 15 species).

Because transmission from dead flies occurs at a high rate, the viral load in dead flies may determine the transmission rate of the virus. Therefore, we checked that our measurements of viral load on day 2 post-infection reflected viral loads on the day of death. We infected 15 species of fly, collected their corpses on the day that they died, and measured their viral load. We found that the viral load on the day of death is tightly correlated to the day 2 viral load (Figure C in S1 Text, inter-specific correlation: 0.84, 95% CI = 0.62, 0.99).

## Discussion

We have observed dramatic variation in virulence when we infected different host species with a viral pathogen. We found both increases and decreases in virulence compared to *D*. *melanogaster*, the host from which the virus was isolated. These changes in virulence were explained by the host phylogeny, with very similar levels of virulence shown by closely related hosts (Fig. 2). Virulence was tightly coupled to viral load (Fig. 4), suggesting the amount of harm caused to a host is a result of virus accumulation in a host, and there is little interspecific variation in tolerance. This in turn means that changes in virulence following host shifts may be accompanied by changes in the transmission rate of the virus.

Large shifts in virulence following pathogen host shifts can result in devastating epidemics, such as myxomatosis in rabbits [19] or Ebola shifting from bats to humans [3]. These changes in virulence often appear random and unpredictable consequences of the pathogen being poorly adapted to its new host [8] or the host being poorly adapted to the pathogen [17]. Our results show that the changes in virulence can be explained by the host phylogeny, with the virus causing similar levels of mortality in closely related species. However, those clades of hosts where virulence is high are scattered across the phylogeny (Fig. 3), so host shifts across large genetic distances may sometimes result in virulent infections and other times benign associations.

DCV was isolated from *D*. *melanogaster* where its virulence is at an intermediate level, and transfer to different species can both increase and decrease virulence. Despite seeing dramatic changes in virulence, there is no tendency for it to decline with genetic distance from the natural host. This contrasts with studies that have investigated infection success in novel hosts and found that it tends to decline in species less closely related to the natural host [29–35]. However, it is difficult to compare our results to these studies as there has been no systematic survey of the host range of DCV, and it may infect species outside of the melanogaster subgroup in nature.

The patterns that we see will depend on the taxonomic scale we are looking at—outside of insects it is unlikely that DCV can infect any hosts [40]. The species we looked at shared a common ancestor 40 million years ago. Combined with the rapid generation time of insects, this makes our results relevant to the taxonomic distances over which hosts shifts normally occur. For example, mammals shared a common ancestor 95 million years ago [72], but have far slower generation times and lower rates of molecular evolution [73,74]

The physiological and molecular reasons why DCV virulence is so variable across Drosophila species are a matter of speculation. A virus’s ability to successfully infect a novel host will depend on its ability to bind to host cells, replicate in suitable tissues and avoid or suppress the host immune response. The ability of viruses to successfully bind to host receptors is important for a successful host shift. For example, changes to the viral capsid of parvoviruses that allow the binding of host cells are essential for them to successfully host shift from cats to dogs [75]. In *D*. *melanogaster* RNAi is known to be an important antiviral immune response [46]; to counter this host immune response, DCV has evolved a suppressor of RNAi that binds dsRNA and inhibits the production of siRNAs [76]. Therefore, the patterns observed may be due to species varying in their ability to produce an antiviral RNAi response, or due to the viral suppressor of RNAi being more or less efficient in different host species, as has been observed for another *Drosophila* virus [77]. In *D*. *melanogaster*, certain alleles of a restriction factor called *pastrel* confer DCV resistance [78], and similar factors may be responsible for the differences in virulence observed between species. The patterns observed could also be due to certain host clades being assayed at sub-optimal environmental conditions (e.g. temperature) that affect immunity or other physiological processes. Whilst we used hosts without *Wolbachia* endosymbionts [47,54], other components of the host microbiota or unknown viral infections may also affect resistance. Regardless of the cause, these changes have happened in the common ancestor of certain clades, resulting in all the descendant species in different clades having similar viral loads and levels of virulence, resulting in the strong phylogenetic patterns we observe (Fig. 3).

Evolutionary theories of virulence commonly assume a direct link between virulence and pathogen loads, but this assumption has been criticised because virulence is often an indirect effect of the pathogen, such as an inappropriate immune response ([8,79], reviewed in [7]). However, in this system we found virulence to be very tightly correlated with viral load. This suggests virulence is a direct consequence of the damage caused by high parasite burdens, although it could also result from viral virulence factors resulting in the high viral loads.

Because virulence is associated with high viral loads, changes in virulence following host shifts are likely to be linked to changes in the transmission rate of the virus. Indeed, it is difficult to conceive that the million-fold range in viral loads that we observed would not affect transmission, and our limited data on transmission tentatively supports this link (see supplementary information). Therefore, host shifts may result in maladaptive viral loads and low rates of transmission in the new host.

When virulence is coupled to pathogen load it can sometimes create a trade-off—higher viral loads result in a higher rate of transmission per unit time but the host dies faster and transmits for less time—meaning that intermediate levels of virulence may be optimal for the pathogen to maximise its fitness [9]. In our case we found high rates of transmission of DCV from the corpses of dead flies to larvae, suggesting killing the host could actually aid transmission. If the majority of transmission occurs from the corpses of dead flies in nature there will be a strong mechanistic coupling between transmission and virulence [80], suggesting the most successful strategy for DCV to evolve will be to maximise its replication rate at any cost to the host. However, the true optimal virulence for DCV remains a matter of speculation, as in nature it will be affected by host population structure [81], environmental conditions [82] and ecological interactions [83]. Regardless, it is clear that host shifts may result in DCV having maladaptive levels of virulence that result in low rates of onward transmission, and this may prevent the pathogen from becoming established in that species.

In conclusion, we have found that host shifts can result in both large increases and decreases in virulence. The fact that virulence is associated with high pathogen burdens, which in turn may lead to higher levels of transmission, suggests that avirulent host shifts may be unsuccessful. We found virulence was largely explained by the host phylogeny, with clades of closely related hosts displaying similar levels of virulence. While this study suggests there is no clear rule to predict whether a pathogen will be virulent in a novel host, it does suggest that if a pathogen causes high levels of virulence in any given host species, it will typically cause similar levels of virulence in closely related hosts.

## Supporting Information

S1 Text Supplementary information. Supplementary methods.Supplementary results.Supplementary Figure A. Time course of DCV infection in 10 host species. Change in viral load is relative to day 0. Each point represents the mean of 3 biological replicates, with each replicate containing 5 flies on average (range 3–5), error bars show standard errors. Flies were 4–6 days old when infected. *D*. *arizonae* and *D*. *hydei* have only 2 biological replicates for each time point and *D*. *hydei* day 1 has only 1 biological replicate.Supplementary Figure B. Viral load relative to the housekeeping gene *RpL32* measured by qRT-PCR at day 0 and day 2 post infection. Each point is a separate biological replicate, with three replicates of most species at each timepoint.Supplementary Figure C. Correlation between DCV viral load on day 2 post infection and viral load in flies collected on the day. Day 2 data is from main experiment. 15 host species were included. The trend line is estimated from a linear model, and has a slope of 0.96 suggesting there is a ~1:1 relationship between day 2 and day of death viral loads. Error bars show standard errors.Supplementary Table A. Full list of species used. All species are in the genus *Drosophila*, with the exceptions of *Hirtodrosophila duncani*, *Zaprionous tuberculatus* and *Scaptodrosophila lebanonensis* and *Scaptodrosophila pattersoni*. The recipes for the food medium reared on are as follows: banana recipe below, cornmeal recipe below, proprionic recipe below, malt recipe in [30]. All cornmeal and proprionic medium had dried yeast sprinkled onto the surface of the food, other food types did not, unless stated. Food plus mushroom means a piece of peeled *Agaricus bisporus* was placed on the surface of the food. Mean wing length is the length of the IV longitudinal vein from the tip of the proximal segment to where the distal segment joins vein V.
(PDF)

S1 Table Genbank accession numbers of sequences used to infer the host phylogeny. Sequences downloaded from genbank are in blue, sequences generated during this project are in red.
(XLSX)

S1 Dataset (CSV)

S2 Dataset (XLSX)

S3 Dataset (CSV)
