# Supplementary material for: The Causes and Consequences of Changes in Virulence following Pathogen Host Shifts
Source: PLoS Pathog. 2015 Mar 16;11(3):e1004728. doi: 10.1371/journal.ppat.1004728 (PMC4361674; doi:10.1371/journal.ppat.1004728)
Supplement: S1 Text — Supplementary methods.Supplementary results.Supplementary Figure A. Time course of DCV infection in 10 host species. Change in viral load is relative to day 0. Each point represents the mean of 3 biological replicates, with each replicate containing 5 flies on average (range 3–5), error bars show standard errors. Flies were 4–6 days old when infected. D. arizonae and D. hydei have only 2 biological replicates for each time point and D. hydei day 1 has only 1 biological replicate.Supplementary Figure B. Viral load relative to the housekeeping gene RpL32 measured by qRT-PCR at day 0 and day 2 post infection. Each point is a separate biological replicate, with three replicates of most species at each timepoint.Supplementary Figure C. Correlation between DCV viral load on day 2 post infection and viral load in flies collected on the day. Day 2 data is from main experiment. 15 host species were included. The trend line is estimated from a linear model, and has a slope of 0.96 suggesting there is a ~1:1 relationship between day 2 and day of death viral loads. Error bars show standard errors.Supplementary Table A. Full list of species used. All species are in the genus Drosophila, with the exceptions of Hirtodrosophila duncani, Zaprionous tuberculatus and Scaptodrosophila lebanonensis and Scaptodrosophila pattersoni. The recipes for the food medium reared on are as follows: banana recipe below, cornmeal recipe below, proprionic recipe below, malt recipe in [30]. All cornmeal and proprionic medium had dried yeast sprinkled onto the surface of the food, other food types did not, unless stated. Food plus mushroom means a piece of peeled Agaricus bisporus was placed on the surface of the food. Mean wing length is the length of the IV longitudinal vein from the tip of the proximal segment to where the distal segment joins vein V. (PDF) [file ppat.1004728.s001.pdf]

Supplementary information

Supplementary methods

Transmission

Adult-adult transmission

Adult males aged 3-6 days old from 38 species were infected with DCV as described above, left for 24 hours, and then single infected flies were placed in a vial with 10 white eyed *D. melanogaster* flies (5 females and 5 males, aged 1-3 days old) from an isogenic line [1] (DrosDel w^{1118}) which acted as sentinels for infection. These flies were then left for 3 days at 22°C and the infected fly was removed. Any vials where the infected fly had died were excluded. The sentinel flies were tipped onto a fresh vial of food and left for 3 further days at 25°C to allow any acquired virus to replicate, before being frozen and undergoing RNA extractions for qRT-PCR, as described above. Out of the 38 species; 32 had 3 biological replicates, 5 had 2 biological replicates and 1 had 1 biological replicate. For each sample four technical replicates of qRT-PCR were carried out with the DCV primers, but we found a number of samples contained low amounts of viral RNA and were on the limit of detection for the qRT-PCR assay. We therefore took a conservative approach by only classifying the sample as infected if all 4 replicates detected viral RNA. We used these data to define a quantitative and categorical measure of infection. The quantitative measure was simply the average viral load relative to the endogenous control across the replicates with samples where all 4 technical replicates did not detect virus being censored. Censored flies were given a ΔCt value of -20 to -Infinity (we estimate a ΔCt value of -20 is just below the detection threshold, and the sample with the lowest ΔCt value where all 4 technical replicates amplified virus had a value of -19.2). For the categorical measure a set of flies were considered infected if all 4 qRT-PCR technical replicates detected viral RNA.

Adult+parent-offspring transmission

The original vial of medium (in which the infected and sentinel flies and been co-housed) was incubated at 25°C, until a second generation of adult sentinels eclosed. The second generation of sentinel flies were frozen and RNA extractions were carried out for qRT-PCR, as described above. From pilot data, we knew transmission via this route of transmission was rare, so we limited ourselves to assaying 8 species (from four major host clades) with high viral loads (these species sit in the top 33% highest viral loads).

Dead adult- larvae transmission

We also measured transmission from the corpses of flies that had died following DCV infection to embryos/larvae. As the chorion of eggs means that embryos are unlikely to be infected we will refer to this as adult-larval transmission, although we cannot exclude infections of the developing embryo. 4-7 day old males from 15 species were infected as described and placed at 22°C. Flies that died within 24 hours of inoculation were presumed to have died from the inoculation procedure and so were not used. Flies were tipped onto fresh medium every 3 days and dead flies were collected daily. Individual dead flies were placed in a fresh vial of medium and 3μl of eggs collected from a population cage of the isogenic *D. melanogaster* DrosDel w^{1118} line were placed into the same vial. Vials were then placed at 25°C for the development of the eggs of the isogenic sentinel flies. After eclosion, 1-2 day old sentinel flies were frozen and their RNA extracted for qRT-PCR, as described above. Out of the 15 species, 14 had 3 biological replicates and 1 had 2 biological replicates.

In order to assess how viral load on day 2 post infection (from the previous experiment) correlated with viral load on the day a fly died, 3-5 day old flies from 15 species were infected as described above, and dead flies were collected twice daily and frozen at -80°C. Flies that died within 24 hours of inoculation were presumed to have died from the inoculation procedure and so were not used. We carried out 3 biological replicates per species, pooling the dead flies within each replicate. Each replicate consisted of 6 dead flies on average (range

of means per replicate=2-10). To calculate the change in viral load from day 0 to death, we measured the change in viral load for the three biological replicates from a single day 0 sample, for each species.

Statistical analyses of transmission data

The statistical analysis of the transmission data follows the same form as those presented for the main analysis above but using a bivariate formulation, where the two mortality response variables were replaced with a single response variable. In the first set of models the response variable was the categorical measure of infection from sentinel flies and was treated as binary. In the second set of models the response variable was the quantitative measure of infection from sentinel flies and was treated as censored Gaussian. All analyses gave similar results so we present the categorical data only. In the model comparing day 2 and day of death viral loads the response was the viral load and was treated as Gaussian. Due to the number of species sampled in these experiments being small (between 15 and 38 species) there was little power for decomposing inter-specific variation into phylogenetic and non-phylogenetic components. Consequently, we fitted two models to each set of data; a phylogenetic model and a non-phylogenetic model. As we detected no adult+parent-offspring transmission, models were not fitted for these data. Data is included as datasets S2 and S3.

Supplementary Results

Transmission

To understand how virulence might be linked with transmission after a host shift, we compared viral loads in different species with transmission rates. The inter-specific correlation between viral load and adult-adult transmission was 0.20 (95% CI= -0.27, 0.92) and the correlation between dead adult- larvae transmission was 0.94 (95% CI= 0.12, 1.00). Treating the species effects as phylogenetically correlated gave similar estimates with even larger confidence

intervals (adult-adult transmission: 0.44, 95% CI= -0.57, 0.99; dead adult- larvae transmission: 0.95, 95% CI= -0.45, 0.99). Therefore, although the point estimates were all positive the credible intervals were large and we can only tentatively say that a positive relationship between viral load and transmission is likely to exist.

Supplementary figures

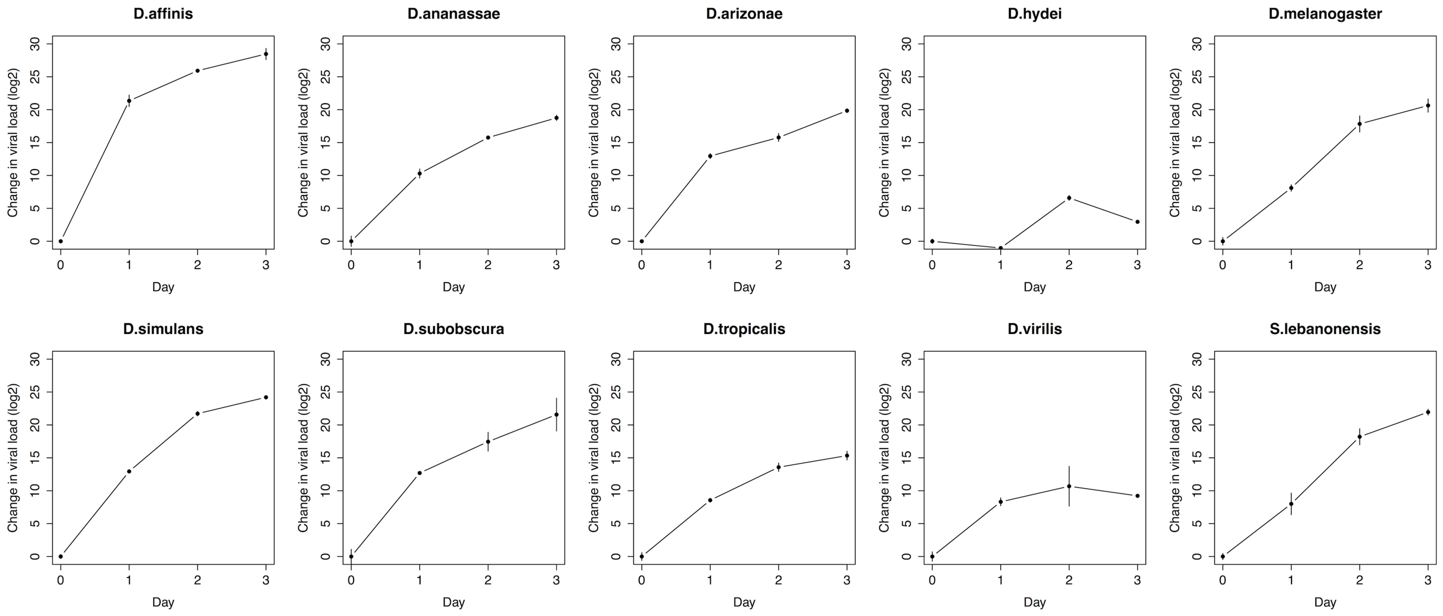

Figure A Time course of DCV infection in 10 host species. Change in viral load is relative to day 0. Each point represents the mean of 3 biological replicates, with each replicate containing 5 flies on average (range 3h 5), error bars show standard errors. Flies were 4h 6 days old when infected. *D. arizonae* and *D. hydei* have only 2 biological replicates for each time point and *D. hydei* day 1 has only 1 biological replicate.

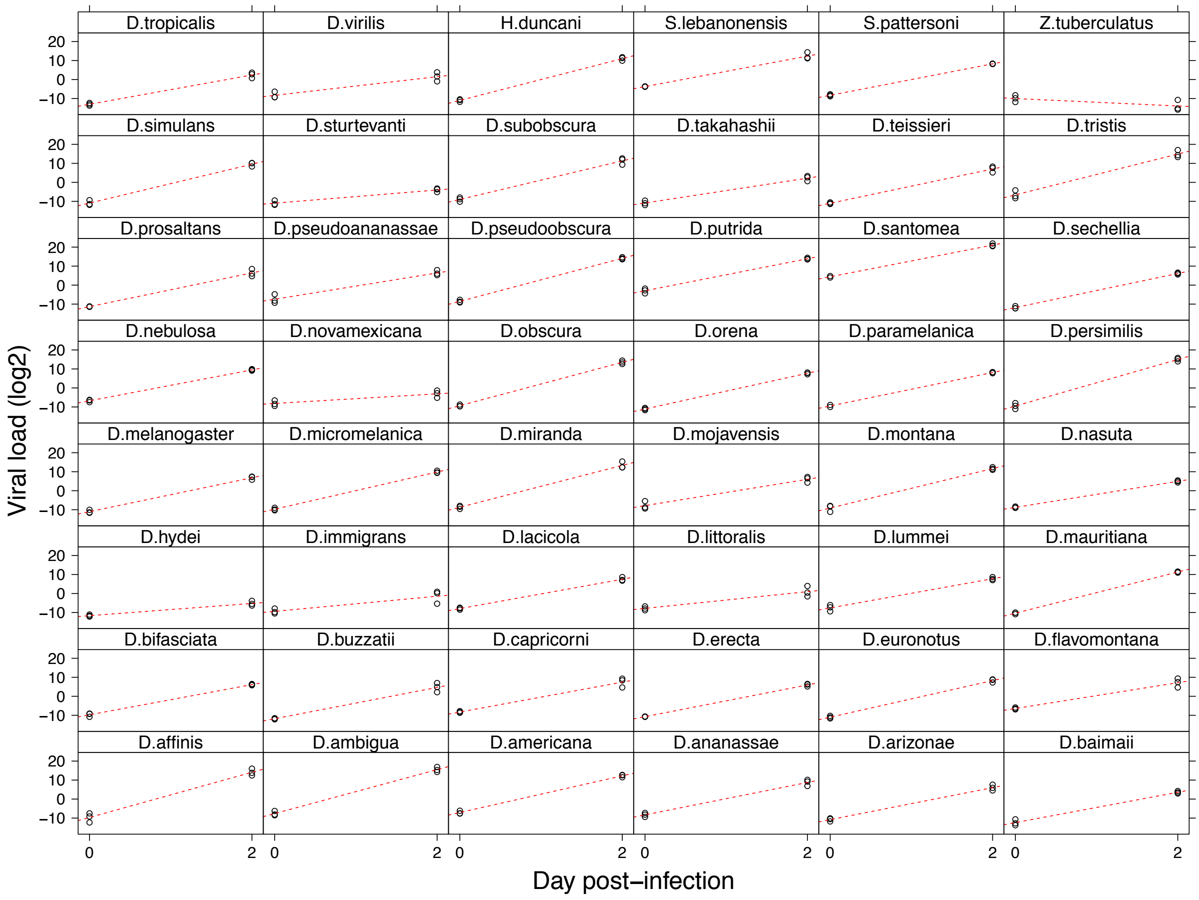

Figure B Viral load relative to the housekeeping gene *RpL32* measured by qRTh PCR at day 0 and day 2 post infection. Each point is a separate biological replicate, with three replicates of most species at each timepoint.

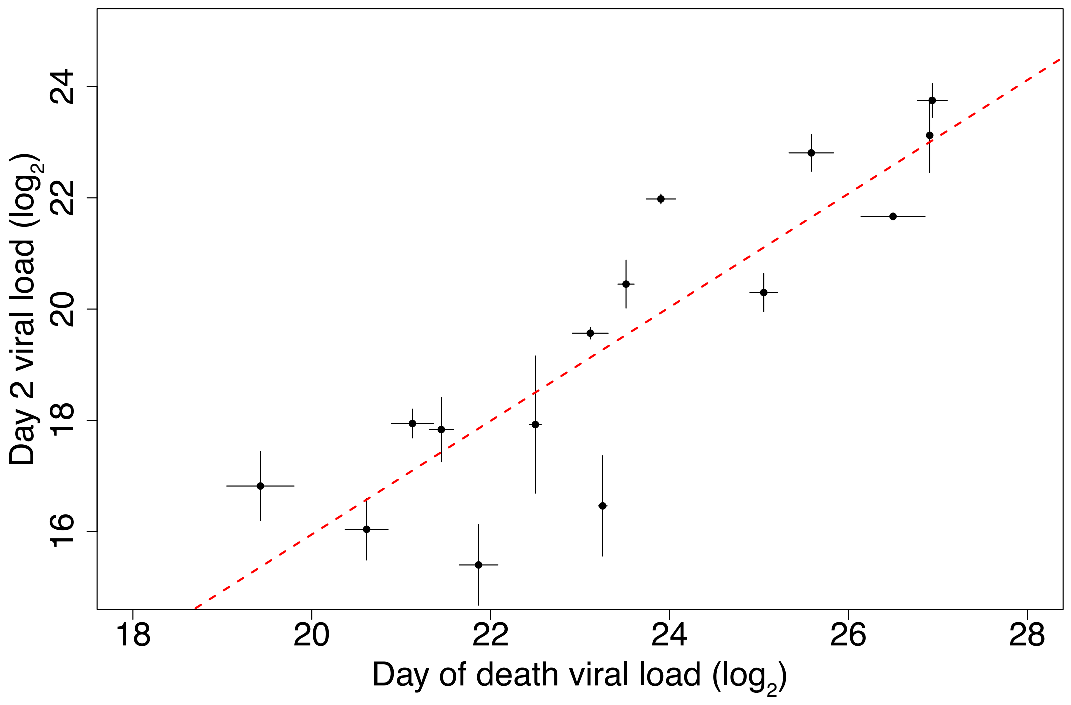

Figure C Correlation between DCV viral load on day 2 post infection and viral load in flies collected on the day. Day 2 data is from main experiment. 15 host species were included. The trend line is estimated from a linear model, and has a slope of 0.96 suggesting there is a ~1:1 relationship between day 2 and day of death viral loads. Error bars show standard errors.

Table A. Full list of species used.

Species	Food	Mean wing length mm
D.affinis	malt	1.706209142
D.ambigua	malt + yeast	2.000294082
D.americana	malt	2.476049427
D.ananassae	cornmeal	1.560527333
D.arizonae	banana	1.586958389
D.baimaii	cornmeal	1.560716162
D.bifasciata	malt	2.046735661
D.buzzatii	malt	1.788852149
D.capricorni	cornmeal	1.909777988
D.erecta	malt + yeast	1.376024216
D.euronotus	cornmeal	2.018607508
D.flavomontana	malt + yeast	2.409073117
H.duncani	proprionic	1.985571654
D.hydei	cornmeal	2.209876743

D.immigrans	malt + yeast	2.300110849
D.lacicola	malt	2.45282603
D.littoralis	banana	2.601529069
D.lummei	malt + yeast	2.424135144
D.mauritiana	proprionic	1.496268456
D.melanogaster	cornmeal	1.774135144
D.micromelanica	cornmeal	1.706285699
D.miranda	banana	2.394762367
D.mojavensis	banana	1.81086324
D.montana	malt + yeast	2.896930399
D.nasuta	cornmeal	1.926682489
D.nebulosa	cornmeal	1.825504323
D.novamexicana	banana	2.43514516
D.obscura	banana + mushroom	2.188772815
D.orena	malt + yeast	1.543053938
D.paramelanica	cornmeal	2.040177065
D.persimilis	malt	1.986954231
D.prosaltans	proprionic	1.634790266
D.pseudoananassae	cornmeal	1.362678733
D.pseudoobscura	banana	1.862782007
D.putrida	proprionic	1.639096979
S.lebanonensis	proprionic	1.892871224
S.pattersoni	Banana	1.905485294
D.santomea	cornmeal	1.418991597
D.sechellia	proprionic	1.452797954
D.simulans	cornmeal	1.436719328
D.sturtevanti	cornmeal	1.774196829
D.subobscura	cornmeal + mushroom	1.968637571
D.takahashii	cornmeal	1.44027451
D.teissieri	cornmeal	1.463215686
D.tristis	banana + mushroom	2.139168449
D.tropicalis	cornmeal	1.540046032
D.virilis	Banana	2.249393421
Z.tuberculatus	banana	1.903543887

All species are in the genus *Drosophila*, with the exceptions of; *Hirtodrosophila duncani*, *Zaprionous tuberculatus* and *Scaptodrosophila lebanonensis* and *Scaptodrosophila pattersoni*. The recipes for the food medium reared on are as follows: banana recipe below, cornmeal recipe below, proprionic recipe below, malt recipe in [2]. All cornmeal and proprionic medium had dried yeast sprinkled onto the surface of the food, other food types did not, unless stated. Food plus mushroom means a piece of peeled *Agaricus bisporus* was placed on the surface of the food. Mean wing length is the length of the IV longitudinal vein from the tip of the proximal segment to where the distal segment joins vein V.

Food recipes

Opuntia Banana

Mixture 1
1000ml water
30g yeast (mix yeast with water, blend for a few seconds)
10g agar (it may be possible to reduce this quantity)
Whisk constantly; bring to the boil for 3-4 minutes.

Mixture 2
20ml Nipagin 150 gms mashed banana
50g corn syrup
30g malt powder
2.5g Opuntia powder.

Add to Mixture 2 to Mixture 1. Whisk constantly and simmer for 5 minutes.

Proprionic

44g Molasses
160g Malt powder
16g Agar
36g Yeast
160g Cornmeal
20g Soya flour
28ml Nipagin
12.4ml Propionic acid
2000ml water

Combine and bring to a boil for 5mins, cool to 70°C before adding Nipagin and Propionic acid.

Cornmeal

1200ml water
13g agar
105g dextrose
105g maize
23g yeast
35ml Nipagin

Combine and bring to a boil for 5mins, cool to 70°C before adding Nipagin

References

1. Ryder E, Blows F, Ashburner M, Bautista-Llacer R, Coulson D, et al. (2004) The DrosDel collection: a set of P-element insertions for generating custom chromosomal aberrations in Drosophila melanogaster. Genetics 167: 797-813.

2. Longdon B, Hadfield JD, Webster CL, Obbard DJ, Jiggins FM (2011) Host phylogeny determines viral persistence and replication in novel hosts. PLoS Pathogens 7: e1002260.
